# Dens invaginatus: a clinical update and a proposed new three-dimensional classification

**DOI:** 10.1038/s41415-026-9617-7

**Published:** 2026-06-12

**Authors:** Lauren Jackson, Polyvios Charalambous, James Darcey

**Affiliations:** 309702228469903784602 University Dental Hospital of Manchester, Manchester, UK; Division of Dentistry, University of Manchester, United Kingdom; 872294006995681332703 University Dental Hospital of Manchester, Manchester, United Kingdom

## Abstract

Dens invaginatus is a developmental anomaly characterised by enamel and dentine infolding, resulting in highly variable internal anatomy that can complicate diagnosis, treatment planning and long-term prognosis. Traditional two-dimensional classifications such as Oehler's Classification do not fully reflect the morphological complexity of invaginations which can now be evaluated with cone beam computed tomography. This paper provides an updated clinical overview of the aetiology, developmental principles, diagnostic features and key considerations for the management of dens invaginatus.

A new three-dimensional classification – the Manchester Classification – is introduced which assesses the vertical extent of the invagination, its horizontal morphology and the proximity to the main canal. This classification offers a more detailed and clinically relevant representation of this anomaly, supporting more predictable diagnostic interpretation and treatment planning.

Management recommendations informed by the Manchester Classification are outlined, offering structured guidance for a range of clinical presentations. The classification is intended to support clinical decision-making and bridge the gap between specialist endodontic understanding and general practice. Early recognition and three-dimensionally informed treatment planning have the potential to improve outcomes for teeth affected by dens invaginatus.

## Key points

- Provides an updated clinical overview of the aetiology, classification and key clinical and radiographic features of dens invaginatus.
- Introduces the Manchester Classification, a new three-dimensional classification system which captures the anatomical complexity of dens invaginatus using CBCT imaging.
- Outlines the recommended management strategies based on the Manchester Classification to support treatment planning for different presentations of dens invaginatus.

## Introduction

Dens invaginatus (DI) is a developmental anomaly characterised by the invagination of the enamel organ into the dental papilla, prior to the completion of calcification in the dental tissues.^1^ This anomaly has been described using various nomenclature including: *dens in dente* by Busch^1^ to denote the radiographic appearance of a tooth within a tooth, dilated composite odontome by Hunter^2^ to describe the abnormal dilation of the dental papilla, gestant anomaly by Colby,^3^ and DI by Hallett.^4^

DI appears the most appropriate as it correctly reflects the pathognomonic infolding of the enamel into the dentine causing the formation of a dead space. This space is an entry point for microorganism colonisation, often only separated from the pulpal tissues by a thin layer of enamel and/or dentine. This makes the invagination more susceptible to caries and, if the malformation exhibits channels to the pulp, it can result in the rapid ingress of bacteria and subsequent pulpal necrosis following the early stages after tooth eruption, emphasising the need for early detection.^1^

## Prevalence

In the permanent dentition, the estimated prevalence of DI ranges from 0.3% to 10%, with the maxillary permanent lateral incisors most commonly affected.^1^^,^^5^ Whilst DI has also been reported in the maxillary central incisors, and less frequently in the maxillary canines and premolars, there have been very few cases reported in the mandible.^6^^,^^7^ In most cases, DI usually presents as a single invagination, but the occurrence of both double and triple forms has been reported, along with the presence of invaginations in supernumerary and primary teeth.^8^^,^^9^ Bilateral involvement of the maxillary lateral incisors has been reported in approximately 43% of cases which highlights the importance of assessing the contralateral tooth, should DI be identified.^10^

## Developmental principles

Although a number of theories regarding the aetiology of DI have been proposed and are outlined in Table 1,^5^^,^^11^^,^^12^^,^^13^^,^^14^ there remains a lack of consensus on the definitive cause of the malformation.

**Table 1:** Summary of the proposed aetiologies for dens invaginatus

**Author**	**Proposed theory of aetiology**
Kronfeld^11^	A failure in the internal enamel epithelium growth whilst the surrounding epithelium continues to proliferate as normal, resulting in the engulfment of the static area and formation of the invagination
Rushton^12^	Rapid proliferation and ingrowth of the internal enamel epithelium into the dental papilla results in the invagination
Atkinson^13^	The external growth pressure on the dental arches results in the infolding of the enamel organ
Bruszt^14^	The ‘twin-theory' where the invagination has formed through the fusion of two tooth germs
Oehler^5^	A result of distortion in the enamel organ during tooth development which results in an enamel-lined channel

Other theories proposed for the development of DI include external factors such as infection^15^^,^^16^ or trauma,^17^ as well as genetic influences where a deficiency of growth molecules involved in enamel organ development can lead to disturbances in the morphology of a developing tooth.^18^ The basis behind the genetic determinants of DI is further supported by the familial tendency of parents and siblings to exhibit similar tooth malformations.^1^^,^^19^ Furthermore, DI has reported associations to other dental anomalies such as microdontia, taurodontism and supernumerary teeth.^20^^,^^21^^,^^22^

## Classification of dens invaginatus

### Oehler's Classification – a two-dimensional classification

**Fig. 1:**
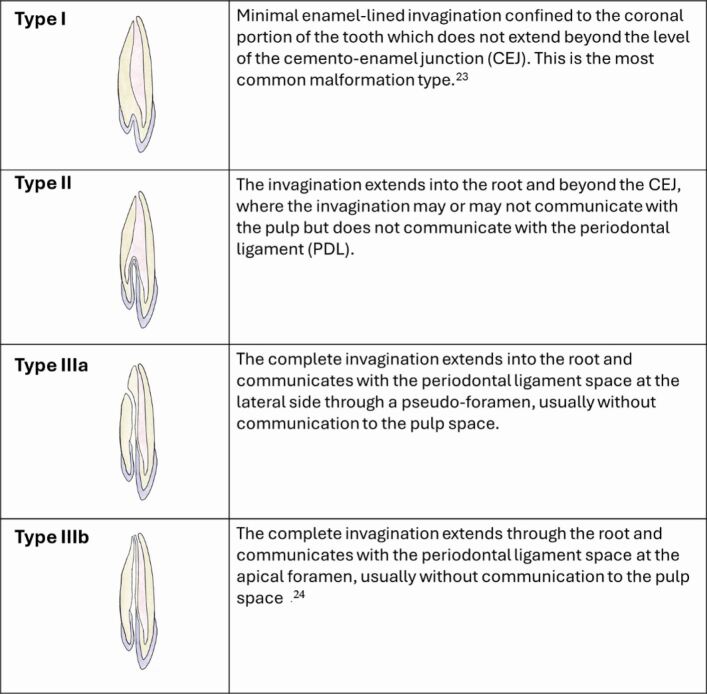
Schematic of Oehler's classification

Dens invaginatus encompasses a wide range of anatomical formations. Generally, DI can be classified according to the radicular and coronal types; since the incidence of radicular DI is very low, Oehler's Classification of coronal DI types is widely accepted in practice.^5^ As seen in Figure 1,^23^^,^^24^ it categorises DI into three distinct groups based on the extent of the invagination and communication with the periapical tissues or periodontal ligament on two-dimensional imaging.

However, this well-known classification has two significant drawbacks:

- The classification was limited by the two-dimensional imaging modalities available at the time of inception
- Conventional two-dimensional imaging limits the ability to fully capture the complexity of the invagination, particularly in the axial plane, which is now readily visualised on three-dimensional imaging.

The increasing use of three-dimensional imaging has already influenced the development of contemporary endodontic classification systems, reflecting a broader shift towards anatomically driven diagnosis and treatment planning. For example, in the context of external cervical resorption, a cone beam computed tomography (CBCT)-based classification that incorporates lesion height, circumferential spread and proximity to the pulp has enabled improved assessment of lesion severity and more predictable treatment planning.^25^ Similarly, a recent three-dimensional pulp canal classification has sought to categorise canal calcification according to the level and extent of canal patency, which can be visualised in greater detail than that previously possible using two-dimensional imaging.^26^ These CBCT-informed classification systems highlight the value of three-dimensional assessment, not only for diagnosis but to help guide clinical decision-making and the selection of appropriate treatment strategies. This supports the rationale for a revised classification of DI that reflects its three-dimensional complexity.

### The Manchester Classification – a new three-dimensional classification

**Fig. 2:**
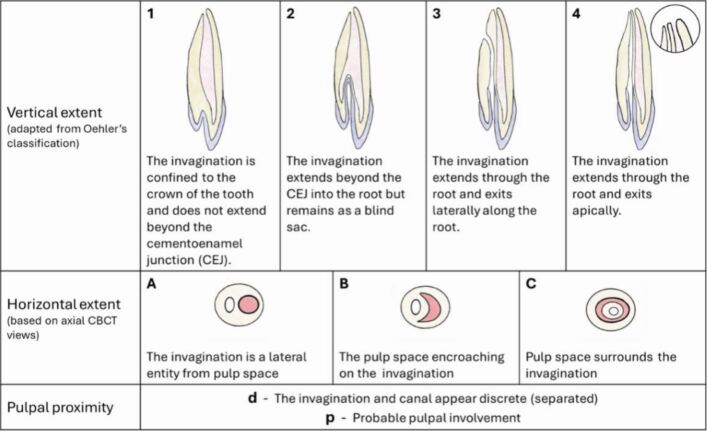
The Manchester Classification based on the three-dimensional anatomy of the invagination

With the growing use of CBCT in the diagnosis and management of DI, there is a new need for a classification system that reflects the three-dimensional nature of this anomaly. The proposed Manchester Classification builds on Oehler's Classification to provide an updated framework designed to assist clinicians in accurately diagnosing and effectively treating these complex cases with respect to both the extent of the invagination vertically and horizontally within the tooth, along with the involvement of the invagination with the main canal (Figure 2).

It is important to recognise that communication with the pulp is often impossible to determine even with modern imaging. The depth and quality of intervening dentine and/or cementum between the invagination and the pulp space will vary from case-to-case, which may explain the development of pulpal necrosis.

This classification defines the invagination according to:

- The vertical extent and point of termination of the invagination (1–4), as described by the well-established Oehler's Classification:
  1. (Oehler Type I): the invagination is confined to the crown of the tooth and does not extend beyond the cementoenamel junction (CEJ)
  2. (Oehler Type II): the invagination extends beyond the CEJ into the root, but remains as a blind sac
  3. (Oehler Type IIIa): the invagination extends through the root and exits laterally along the root
  4. (Oehler Type IIIb): the invagination extends through the root and exits apically
- The horizontal extent of the invagination (A-C), which can be observed on the axial CBCT view to describe the location of the invagination and interposition of the pulp and invagination:
  A. The invagination is a separate entity from the pulp space
  B. The invagination encroaches on the pulp space
  C. The invagination is surrounded by the pulpal space
- The pulpal proximity (d or p) of the invagination to the pulp space, which can be decided based on the axial CBCT view in combination with pulpal testing and presence of pathology associated with the tooth.

(d). The invagination is a discrete entity from the main canal

(p). The invagination is likely involved with the main canal.

Together, these parameters offer a more comprehensive and detailed characterisation of the DI three-dimensionally and will therefore be used throughout this paper to discuss the diagnosis and treatment for DI.

## Diagnosis of dens invaginatus

### Clinical presentation

**Fig. 3:**
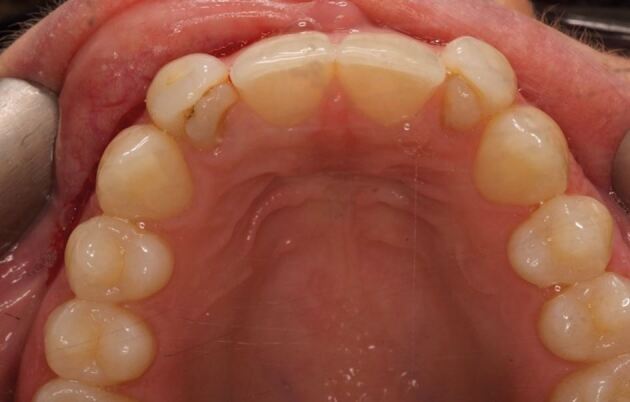
Unusual inclusions or pits on the palatal surface are often an early indicator for the presence of a dens invaginatus

**Fig. 4:**
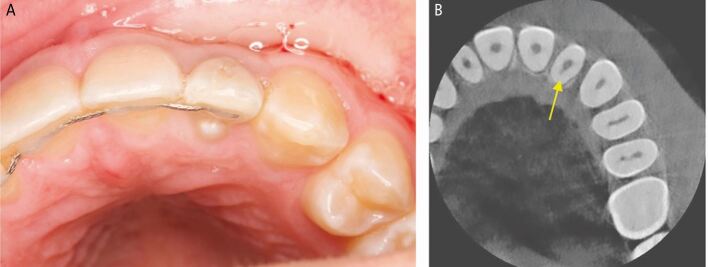
More often the evidence of an invagination is more subtle, and necrosis of an otherwise healthy, non-restored and non-traumatised tooth is often the only signpost to a dens. (A) The enamel projection on the palatal aspect of the maxillary left lateral incisor disguised the dens invagination. (B) Axial CBCT shows a thready pulpal connection (yellow arrow)

The clinical appearance of DI can vary considerably but early diagnosis is important for the prognosis of the tooth. The crown can have normal morphology with no evidence of malformation or, more often, it exhibits abnormal crown morphology including peg-shaped or barrel-shaped form, increased mesiodistal and/or labial-lingual diameter, or the presence of a talon cusp, deep palatal pit or labial groove (see Fig. 3 and Fig. 4).^27^^,^^28^^,^^29^ The presence of a deep foramina caecum on the palatal or occlusal surface of the tooth is commonly present as the entrance of the invagination.

### Radiographic presentation

Teeth affected with DI can be an incidental finding on a radiograph following the exacerbation of clinical signs and symptoms. If an invagination is suspected, periapical radiographs are the standard choice of two-dimensional imaging. It may be advisable to consider undertaking two periapical radiographs in parallax with at least a 15° change in horizontal angulation to ensure the lesion is not missed.^1^^,^^30^ The invagination will usually appear as a radiopaque area, similar in radiodensity to that of enamel, with a central radiolucent core that extends from the cingulum or incisal edge to varying depths near or within the pulp space. The CEJ is used as a boundary to differentiate type 1 to 2 invaginations, as the latter will cross the CEJ and extend as a pocket into the root, but not reach the apical portion or connect with the periodontal ligament (PDL). More vertically extensive lesions can appear as a deep enamel-lined fissure with complex root canal anatomy communicating into the periodontal ligament, either through the lateral aspect of the tooth (type 3) or the apex (type 4).^31^ If subsequent pulpopathologies develop, an atypical ‘butterfly-like' radiolucency in the periapical region will be seen which corresponds to the two sources of inflammation, i.e., the pulp and invagination.^1^

**Fig. 5:**
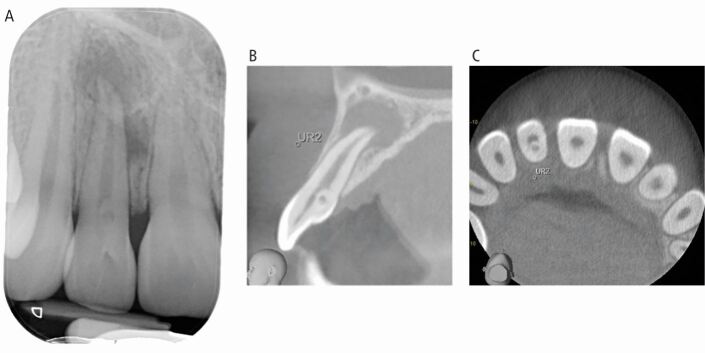
2Bp dens invaginatus. (A) Pre-operative radiograph reveals chronic periapical periodontitis of the maxillary right lateral incisor with a dens invaginatus anomaly. (B) Localised CBCT was recommended due to the depth of the invagination and the sagittal view shows the invagination extending from the palatal pit into the coronal third of the root, where it communicates with the root canal. (C) Axial CBCT view reveals partial encroachment of the invagination on the main canal

**Fig. 6:**
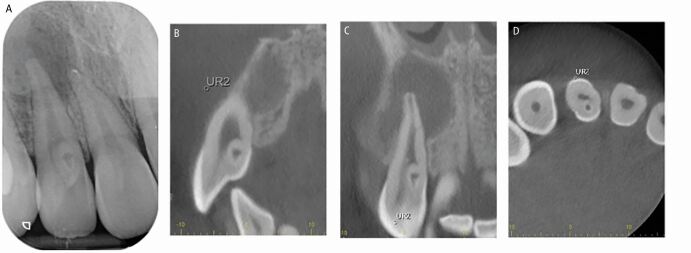
2Bp dens invaginatus. (A) Pre-operative periapical radiograph demonstrates a diffuse periapical radiolucency associated with the maxillary right lateral incisor and a dens malformation. (B) Sagittal CBCT confirms the invaginatus as a blind ending and enamel lined sac coronal to the cementoenamel junction. (C) Coronal CBCT shows smooth remodelling of the palatal surface of the apical half of the root, suggestive of external inflammatory resorption. (D) Axial CBCT view shows the main canal encroaching on the invagination with a probable communication between the two

Once the provisional diagnosis is made, CBCT is the established imaging modality to confirm and capture highly detailed three-dimensional imaging of the invagination. This will reveal the type and extent of the invagination to aid in the diagnosis and develop an appropriate management plan for the more complicated cases (Fig. 5 and Fig. 6).^23^ Owing to the increased radiation dose, CBCT must be reserved for cases where pathology presents, and a definitive plan is necessary.^32^ The type of scanner and scanning protocol must be considered since the use of CBCT for endodontic purposes requires specific acquisition parameters that differ from those used for general dental or orthodontic imaging. Specifically, the use of a small field of view and high-resolution imaging is essential to accurately assess the complexity of the invagination and its relationship to the main canal.^33^

## Treatment strategies for coronal dens invaginatus

Treatment strategies for DI in permanent teeth vary according to:

- The vertical and horizontal extent of the invagination
- The complexity of the invagination
- The relation of the invagination to the main canal
- The presence of a communication between the invagination and PDL
- The pulpal health of the canal
- The infective status of the invagination
- The morphology of the apex
- The aesthetics of the tooth.

When determining the most appropriate treatment approach, the pulpal status of the tooth and the infective status of the invagination are key factors to guide the initial management. Once pulpal status has been established, evaluation of the vertical and horizontal extent of the invagination on three-dimensional imaging is required to allow the formulation of a definitive treatment plan. Structured treatment guidance based on the Manchester Classification is presented in Table 2.

**Table 2:** Treatment recommendations for dens invaginatus clinical scenarios using the proposed Manchester Classification, to illustrate how three-dimensional anatomy may inform clinical decision-making

	**Classification subtypes**	**Recommended management**
No pathology	All	Seal with resin composite and monitor
Caries within the invagination	All	Remove caries; anticipate need for vital pulp therapy; seal with resin composite and monitor
Contamination of invagination space only (d)	A (invagination discrete entity from pulp space)	RCT of invagination only
	B (pulp space encroaching on invagination)	
	C (pulp space surrounds invagination)	
Necrosis of main canal space (p) (possibly in all types of vertical extension 1-4)	A	RCT of pulpal space only
	B	RCT of pulpal space only; maximise chemical debridement and use warm vertical condensation technique to clean and fill space
	C	Consider removal of invagination entirely (if feasible) and RCT of remaining space only OR consider apical surgery/extraction
Necrosis of both canal space and invagination	A	RCT both canal and invagination; treat as separate ‘canals'
	B	RCT both canal and invagination; maximise chemical debridement and use warm vertical condensation technique to clean and fill space. Anticipate that enlargement of the invagination may perforate into pulp canal space and vice versa
	C	Consider removal of invagination entirely if feasible and RCT of remaining space only OR consider apical surgery/extraction
**Other considerations**		
Aesthetics	Teeth with dens invaginatus may appear larger and more tuberculate. In unilateral cases with asymmetry of tooth shape, consider: Extraction and orthodontic treatment to balance the spaces Orthodontic opening of the contralateral space to allow restorative camouflage of the more diminutive tooth. Where aesthetics are significantly compromised and the invagination is complex (3B, 3C, 4B, 4C), extraction may offer the more predictable outcome and should be discussed with the patient	
Vertical extent of the invagination	1 or 2: typically more straightforward; the invagination is a deep pit in the crown or coronal canal. Obliteration is often possible, and though access may be destructive, subsequent RCT is simpler 3 or 4: More complex and challenging; obliteration of the invagination is time-consuming and risks perforation. CBCT assessment is essential to understand pulp–invagination relations	
Apex morphology	For immature roots or open apices of the main canal (or invagination), if possible consider: Apexification with a bioceramic material or pulp revascularisation techniques Surgical apical plug in cases with more complex vertical/horizontal anatomy	
CBCT, cone bean computed tomography; RCT, root canal treatment		

If the pulp is healthy, the clinician may only need to focus their attention on the invagination. If the pulp is not healthy, intervention by means of non-surgical endodontic treatment is required. This treatment is often complex owing to the altered internal anatomy associated with the invagination and may be further complicated by the intimacy of the invagination and the canal along with any disturbances to root development.^34^ Where the root of the affected tooth is immature or the apical foramen of the main canal (or the invagination) is ‘open', treatment may include:

- An apical barrier technique with a bioceramic material
- Pulp revascularisation techniques
- In more complex cases: a surgical approach to the tooth.^24^

Considering the vertical extension of the invagination, generally type 1 and 2 invaginations are relatively more straightforward to manage as the invagination is essentially a deep pit into the crown or coronal root. Sealing or obliteration of the invagination is often feasible and, though the access becomes more destructive, the ensuing root canal treatment becomes simpler. Type 3 and 4 invaginations are inherently more challenging, and obliteration of the canal may be time-consuming and bring with it the risk of perforation. CBCT scans should be meticulously reviewed to determine the relationship of the pulp to the invagination (see Table 2).

### Absence of caries and pulpal pathology

In the absence of clinical or radiographic evidence of caries or pulpal disease, typically seen in type 1 and 2 invaginations (but could be all types), the invagination can be protected through placement of flowable composite resin over the entrance of the malformation to reduce the risk of caries development. If the entrance of the invagination is not clinically visible, it is important to still seal the pits and fissures to prevent bacterial contamination and ensure that there is a long-term follow-up plan in place.

For type 3 and 4 invaginations, three-dimensional imaging is essential as these represent the most complex form of the anomaly and present challenges in accurately determining the pulpal vitality of the tooth. Where the tooth is asymptomatic and shows no evidence of pulpal pathology, it is recommended to seal the invagination with flowable composite resin as above. Instrumentation should be avoided as the defect will be in close proximity to the main root canal system, and prophylactic access and debridement of these invaginations would likely result in iatrogenic pulpal exposure.

### Caries within invagination without pulpal involvement

**Fig. 7:**
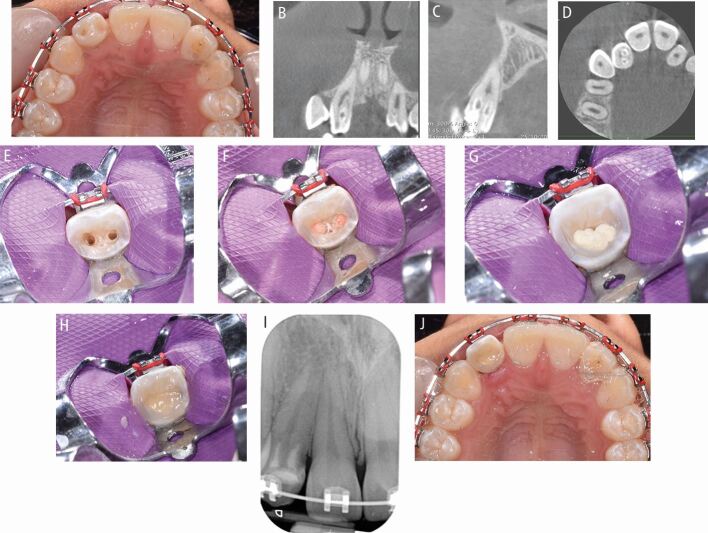
2Ad double dens invaginatus. (A) Clinical presentation of carious invaginations associated with the maxillary right lateral incisor. (B) Coronal CBCT (C) and sagittal CBCT confirm two enamel lined pits. (D) Axial CBCT view demonstrates the invaginations have no communication with the main canal. (E) Each invagination was separately accessed and instrumented. (F) The invaginations were filled with thermoplastic gutta percha, leaving the main canal undisturbed. (G) Bioceramic putty was placed over the pulpal area to encourage hard tissue formation. (H, I) show resin composite placed as the final restoration. (J) Final post-operative radiograph

**Fig. 8:**
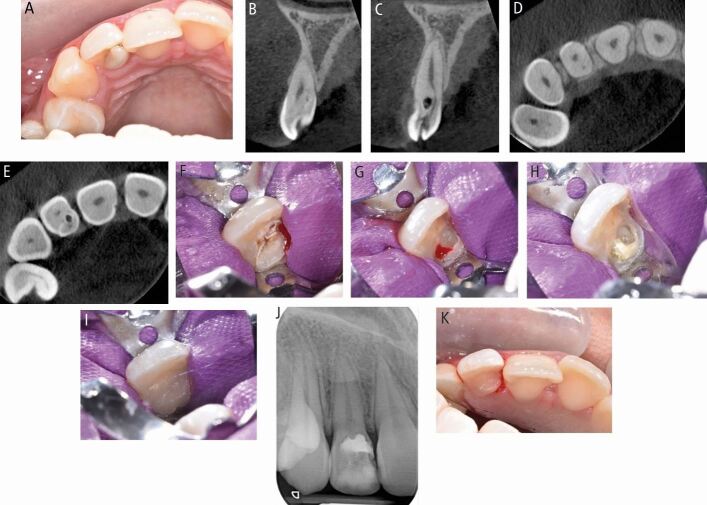
2Bp invagination of a vital maxillary right lateral incisor. (A) Carious palatal invagination of a maxillary right lateral incisor. (B, C) Sagittal CBCT views clearly shows the vertical extension of the invagination beyond the cementoenamel junction, i.e., type 2. (D, E) Axial CBCT views suggests partial circumferential extension of the pulpal space and encroachment on the invagination, i.e., type B. (F) Initial exploration of the invagination identified a direct communication of the invagination with the pulp, i.e., ‘p'. (G) Cvek pulpotomy was performed. (H) Bioceramic hydraulic calcium silicate cement used as a liner and (I) restored with composite. (J) Final post-operative radiograph. (K) Clinical presentation following treatment

Types 2, 3 and 4 invaginations represent more extensive defects which can extend towards the main pulp space. If caries develops within these invaginations, it can advance deeply whilst remaining clinically undetectable. Where the main pulp remains vital, treatment should be confined to the invagination. It is recommended to instrument the coronal opening of these invaginations to allow visualisation and debridement of the defect using sodium hypochlorite, without disturbance to the main root canal system. As these invaginations are in close proximity to the root canal system, it is advised to use bioceramic materials to promote hard tissue formation and provide a definitive restoration in composite resin (Fig. 7, Fig. 8).

### Contamination of the invagination

Peri-invaginatus periodontitis,^35^ typically developing from type 3 and 4 lesions, presents when the invagination becomes necrotic and inflammation presents in the periodontal tissues, whilst the main root canal system remains vital and healthy.^36^ This condition requires debridement and obturation of the invagination, in a manner similar to root canal treatment, with every effort made to preserve the pulp vitality through treatment of the invagination in isolation. This treatment approach may only be feasible with a type 3 invagination as the apical proximity of type 4 invaginations would make this challenging. The entire invagination should be incorporated into the access cavity, taking care to avoid unnecessary disturbances to the main canal, as the invagination will be lined with enamel and cementum. The use of magnification with co-axial illumination is essential to facilitate careful enlargement, often requiring ultrasonic tips and Gates Glidden burs to ensure sufficient instrumentation and utilisation of activated irrigation to improve the efficacy of debridement.^37^^,^^38^ As the invagination communicates with the periodontal ligament space, obturation using a bioceramic material is recommended to promote healing of the peri-radicular tissues.

When orthograde treatment fails or the invaginated canal cannot be adequately cleaned and filled, surgical management may be necessary.^8^ Extraction remains the final option when endodontic treatment, surgery, or a combination of both proves unsuccessful.

### Pulpal necrosis of the main canal system

**Fig. 9:**
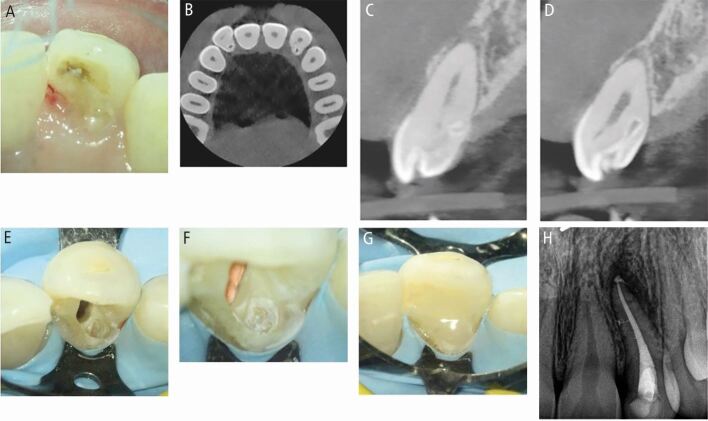
Necrotic maxillary left lateral incisor with a 2p invagination. (A) Caries evident and a separate unusual palatal inclusion can be seen as a blind sac. (B) Axial CBCT reveals the maxillary left lateral incisor invagination as a distinct entity and a dens invaginatus of the contralateral lateral incisor. (C, D) Sagittal CBCT views confirms apical pathology associated with the maxillary left lateral incisor and hence pulpal involvement. (E) The main canal accessed, cleaned and shaped. (F) The main canal was filled with gutta percha whilst the invagination was sealed separately using composite. (G) Maxillary left lateral incisor fully restored with composite. (H) Post-operative radiograph

If pulpal necrosis of the main canal is evident, orthograde endodontic treatment is required. For (1–4)A invaginations, root canal treatment of the main canal should be undertaken whilst leaving the invagination undisturbed (see Fig. 9). For (1–4)B invaginations, provided no communication is suspected between the main canal and the invagination (d), root canal treatment should be limited to the main canal alone; however, chemo-mechanical debridement must be optimised due to the proximity of the main pulp to the invagination and obturation techniques such as warm vertical compaction^39^ or hydraulic condensation^40^ used to ensure optimal sealing of the canal. For (1–4)C invaginations, i.e., complete or almost complete enclosure of the invagination by the main canal, removal of the entire invagination will be necessary to facilitate root canal treatment of the main canal since it is encompassed by it. Owing to the complex nature of this anatomy, if orthograde treatment fails, surgical management with an apical plug or even extraction of the tooth may be required.

### Pulpal necrosis of both the invagination and the main canal system

In cases where necrosis affects the main canal and the invagination, management will depend on the circumferential relationship between these two structures. In (1–4)A invaginations, where the canal and invagination are discrete entities (d), both should be treated endodontically using thermoplastic gutta percha to ensure the complex anatomy is fully sealed, whilst maintaining them as separate entities. In cases where the main canal presents with an immature root and an open apex, apexification^35^^,^^41^ or regenerative endodontic procedures^42^ may be considered (see Fig. 9).

**Fig. 10:**
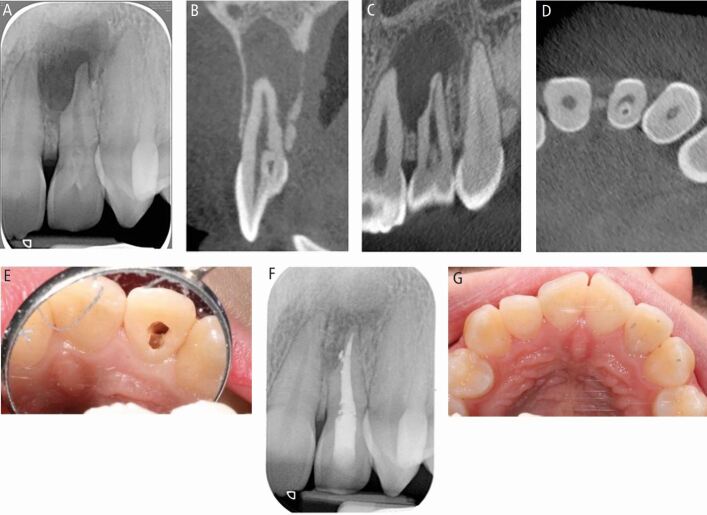
2Bp invagination with subsequent necrosis. (A) Pre-operative radiograph confirms periapical pathology associated with the maxillary left lateral incisor. (B) Sagittal CBCT confirms the vertical extension of the invagination beyond the cementoenamel junction. (C) Coronal CBCT and (D) axial CBCT shows the extent of the invagination and relationship between the invagination and main canal. (E) Root canal treatment was initiated, ensuring the invagination was incorporated into the access cavity preparation. (F) Radiographic outcome after full shaping, cleaning and obturation was completed. (G) The maxillary left lateral incisor was restored using composite

For (1–4)B invaginations, where the pulp space is encroaching upon the invagination, orthograde root canal treatment of both canals remains the treatment of choice, at least initially unless during the instrumentation of the two results in them merging together. An emphasis must be placed on maximising chemo-mechanical debridement and adopting thermal obturation techniques (see Fig. 10).

**Fig. 11:**
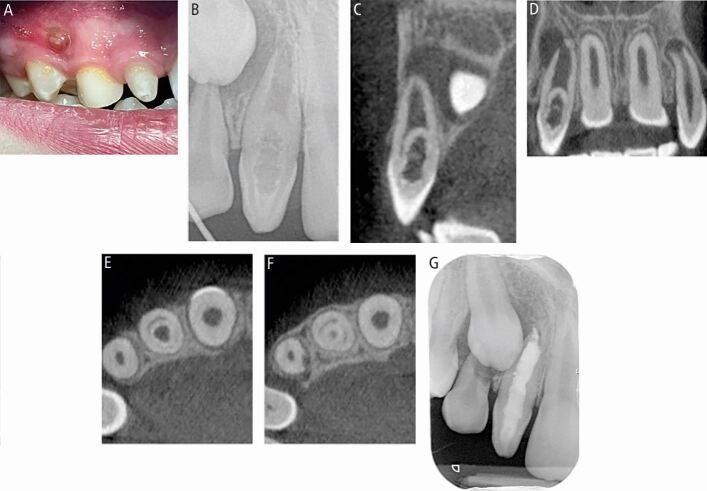
2Cp invagination. (A) Clinical presentation of a ten-year-old presenting with a sinus distal to the right maxillary deciduous canine and conical lateral incisor. (B) Periapical radiograph confirms likely source of pathology is wide type 2 invagination associated with the maxillary right lateral incisor. (C) Sagittal CBCT confirms extent of the invagination into the coronal third of the root. (D) Coronal CBCT confirms no invagination associated with the maxillary left lateral incisor. (E, F) The pulp space encompassing the invagination, i.e., type C. (G) Final post-obturation radiograph following removal of the entire invagination and root canal treatment using an apical plug and warm vertical compaction techniques

In (1–4)C invaginations, where the pulp space completely (or almost completely) surrounds the invagination and pulpal involvement is probable (p), removal of the entire invagination should be considered if feasible, followed by endodontic treatment of the main canal space (see Fig. 11). In cases where this approach is not possible, surgical endodontics or even extraction may be required.

### Aesthetic considerations

Teeth with DI may present with abnormal crown morphology that can compromise dental aesthetics, with common presentations including being larger or more tuberculate in shape, conical or peg-shaped forms, barrel-shaped or bifid crown configurations, or an exaggerated or prominent cingulum.^43^ However, it is important to recognise that more than 40% of affected teeth may present with a clinically normal crown morphology.^44^^,^^45^

As mentioned above, maxillary lateral incisors are the teeth most commonly affected DI, but DI on other anterior maxillary teeth is not unheard of.^1^ Being prominent in the smile zone, these teeth contribute substantially to overall facial aesthetics since the altered crown morphology can result in noticeable asymmetry when the condition is unilateral. In cases where the invagination is unilateral with significant asymmetry of the crown form, treatment approaches vary based on the severity of aesthetic compromise. For mild to moderate concerns, multidisciplinary treatment combining endodontic with restorative procedures such as restorative camouflage with resin composite or veneers can yield successful outcomes. Where DI teeth appear more tuberculate with an increased mesiodistal width, orthodontic treatment to open the contralateral space and allow restorative camouflage of the more diminutive tooth should be considered.

However, for complex invaginations (particularly types 3–4 combined with B–C anatomy) where aesthetic compromise is significant, extraction often represents the more predictable treatment outcome. Following extraction, orthodontic space closure or opening of the contralateral space and restoring the bilateral spaces can facilitate aesthetic and functional rehabilitation. The decision between conservative management, extensive restorative therapy, or extraction should be guided by the invagination type and complexity, degree of aesthetic compromise, patient age, treatment prognosis and patient preferences.

## Radicular dens invaginatus

**Fig. 12:**
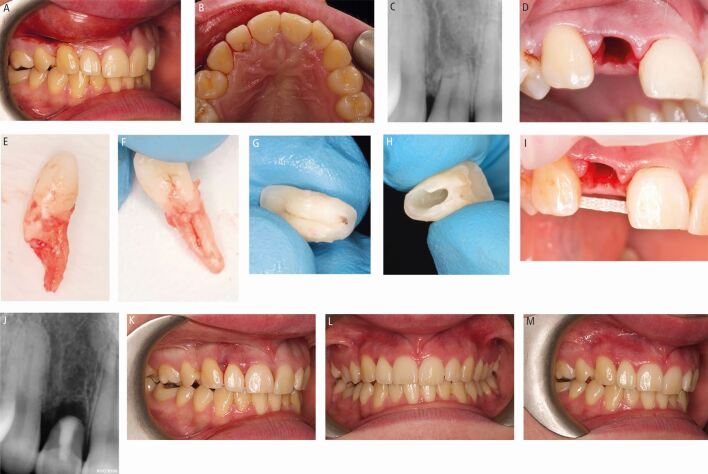
A radicular variant of dens invaginatus. (A) Maxillary right lateral incisor appears with normal morphology apart from (B) a deep root groove on the palatal aspect. (C) Pre-operative radiograph displays an infection which is deemed uncontainable. (D) Maxillary right lateral incisor was removed. (E, F) Differing perspectives of the invagination. (G, H) Maxillary right lateral incisor following amputation of the invagination and prior to filling with glass ionomer cement. (I) Re-attachment of maxillary right lateral incisort was undertaken using fibre-reinforced composite. (J) Post-operative radiographs shows the coronal aspect of the root *in situ* following treatment with glass ionomer cement. (K) Maxillary right lateral incisor immediately after re-attachment. (L) Anterior view of tooth maxillary right lateral incisor and (M) lateral view at 12 months post-operatively

Root grooves have been documented as a finding alongside DI, most commonly within the radicular variants. Radicular DI is a rare dental anomaly, originating from the infolding of Hertwig's epithelial root sheath into the root after crown development has been completed.^46^ The invagination is confined to the root and can either be cementum-lined and associated with an axial root groove, or enamel-lined within the root.^47^ Various treatment modalities have been considered for radicular DI including intentional replantation, but when radicular DI is present, the prognosis for the tooth must be lowered appropriately as external biofilm control may be impossible (see Fig. 12).

## Conclusion

DI presents in a wide range of clinical and radiographic forms and there are a number of treatment approaches to treat this defect, based on the type of invagination and pulpal status of the tooth. The Manchester Classification attempts to acknowledge the complexity of DI in three-dimensions using CBCT assessment and it is hoped this will help the clinical team understand DI, improve diagnosis of DI and articulate and deliver management more confidently. Early diagnosis and preventative treatment will improve the prognosis for teeth affected with DI by allowing preservation of healthy pulp and preventing the need for complex endodontic treatment. When endodontic therapy is indicated, the advent of three-dimensional imaging has significantly enhanced the efficiency of diagnosing and managing cases of DI. Nevertheless, careful consideration must be given to the risks and benefits of each treatment modality, with a strong emphasis on preserving pulp vitality and selecting the least invasive approach whenever possible.
